# Prediction of Elastic Modulus of Leached Fly Ash Concrete Based on Non-Uniform ITZ Model

**DOI:** 10.3390/ma18163779

**Published:** 2025-08-12

**Authors:** Xiaoping Zhao, Misha Zhan, Zhiwei Chen, Jian Zhang, Qiang Li, Wenbing Song

**Affiliations:** 1 Architectural Engineering College, Jinhua University of Vocational Technology, Jinhua 321016, China; jgqszxp@163.com; 2 Jiyang College, Zhejiang A&F University, Shaoxing 311800, China; jianzhang_zjyc@163.com; 3 School of Civil Engineering and Architecture, Zhejiang University, Hangzhou 310058, China; 4 College of Design and Engineering, National University of Singapore, 21 Lower Kent Ridge Rd, Singapore 119077, Singapore; zhiweichen1994@163.com; 5 Keyi College, Zhejiang Sci-Tech University, Shaoxing 312369, China; lq527885541@hotmail.com

**Keywords:** calcium leaching, fly ash concrete, elastic modulus, composite sphere model, non-uniform interfacial model

## Abstract

The incorporation of fly ash into concrete reduces cement consumption by 10–30%, lowers CO_2_ emissions by 30–50%, cuts costs by 15–25%, and enhances durability, thus reducing maintenance expenses. However, the predictive model for the elastic modulus of fly ash concrete subjected to calcium leaching is still lacking. Regarding the theoretical method, the content of calcium hydroxide and calcium silicate hydrate in fly ash–cement systems is quantitatively calculated according to the hydration reaction relationship between cement, fly ash, and water, and then the porosity of the fly ash–cement matrix and interface transition zone (ITZ) after calcium leaching can be obtained. Based on the theory of two-phase composite spheres and the non-uniform ITZ model, the prediction method for the elastic modulus of leached fly ash concrete can be constructed, which comprehensively considers key parameters such as fly ash content, non-uniform characteristics of the ITZ, and the water–binder ratio (w/b). Additionally, the corresponding experimental investigation is also designed to study the variation regulation of the leaching depth, leaching extent, and elastic modulus of fly ash concrete with leaching time. The prediction method for the elastic modulus of leached fly ash concrete is validated via self-designed experimental methods and third-party experiments. This study further delves into the specific effects of w/b, aggregate volume fraction (f_a_), fly ash content, and ITZ thickness (h_ITZ_) on the elastic modulus of leached concrete (E). The research findings indicate that an appropriate amount of fly ash can effectively enhance the leaching resistance of concrete. For a leaching degree of 10.0%, 30.0%, and 50.0%, E at w/b = 0.40 exceeds that of w/b = 0.60 by 26.71%, 28.43%, and 30.28%, respectively; E at h_ITZ_ = 10 μm exceeds that of h_ITZ_ = 50 μm by 16.96%, 15.80%, and 15.11%, respectively; and E at f_a_ = 65% is 39.82%, 43.15%, and 46.12% higher, respectively, than that of concrete with f_a_ = 45%. Furthermore, a linear correlation exists between the elastic modulus and the degree of leaching. The prediction method for the elastic modulus offers a theoretical foundation for in-depth exploration of the durability of leached mineral admixture concrete and its scientific application in practical engineering.

## 1. Introduction

Concrete structures such as dams, nuclear power plants, underground tunnels, and harbors, subjected to long-term exposure to soft water immersion, face severe threats from calcium leaching [1,2], a complex and highly detrimental process. Under prolonged exposure to soft water, the solid-phase calcium within the hydration products of concrete gradually dissolves and leaches out. This process not only modifies the internal microstructure of concrete, leading to the expansion of the pore structure and an increase in porosity, but also significantly compromises the durability and mechanical properties of concrete structures [3,4]. From a mechanical perspective, the elastic modulus, serving as a critical indicator for evaluating the overall stiffness and deformation capacity of concrete structures, exhibits a significant reduction during calcium leaching. This reduction renders the structure more susceptible to deformation under load, thereby adversely affecting its safety and structural stability [5,6]. Regarding durability, calcium leaching accelerates concrete deterioration and diminishes its resistance to external environmental attacks. This elevates the risk of penetration by harmful ions, potentially triggering a cascade of issues such as reinforcement corrosion, consequently drastically shortening the service life of concrete structures [7,8,9].

To enhance concrete durability, fly ash, one of the most widely used mineral admixtures, is extensively incorporated into concrete mix designs. Characterized predominantly by spherical glassy particles and pozzolanic activity, its inclusion not only effectively reduces cement consumption and carbon emissions but also significantly improves the overall properties of concrete [10,11]. Specifically, the pozzolanic reaction of fly ash consumes calcium hydroxide (CH) formed during cement hydration, generating hydration products like calcium silicate hydrate (C-S-H). This process enhances concrete compactness and durability. Furthermore, through its micro-aggregate effect, fly ash fills internal pores, optimizes the microstructure, and reinforces erosion resistance [12,13]. Nevertheless, the mechanism by which calcium leaching influences the elastic modulus of fly ash concrete in leaching environments remains unclear, necessitating urgent in-depth investigation to improve engineering practice. In concrete materials, the volume fraction and particle size of aggregates exert significant influences on the elastic modulus of concrete. Relevant research results indicate that the aggregate volume fraction is positively correlated with the elastic modulus. Due to the higher elastic modulus of the aggregates, the deformation resistance of the concrete is significantly enhanced with the increase in the volume fraction of the aggregate, leading to an increase in elastic modulus. The influence of aggregate particle size on the elastic modulus is complex. An appropriate particle size can form a rational skeleton structure, facilitating stress transfer and thereby enhancing the elastic modulus, whereas excessively large or small particles can reduce the elastic modulus due to induced structural imperfections [14]. In comparison, the aggregate shape has a relatively minor influence on the mechanical properties and elastic modulus of concrete. It is noteworthy that the extent of this influence is significantly less pronounced than that of the aggregate volume fraction and particle size [15]. The influence of the cement paste matrix on the elastic modulus of concrete primarily depends on two factors: the water–cement ratio and the type of cement. Among these, the water–cement ratio is a key parameter affecting the microstructure of the cement paste. A lower water–cement ratio can markedly improve the paste’s microstructure. For instance, when the water–cement ratio drops from 0.5 to 0.4, the porosity of the paste is substantially reduced, its compactness is significantly enhanced, and the elastic modulus of the concrete increases correspondingly by approximately 20–30% [16]. Additionally, using high-grade cement or incorporating highly reactive mineral admixtures can optimize the structure of hydration products, making them denser and thereby further enhancing the stiffness of the cement paste [17]. The ITZ, the weak area between aggregates and cement paste in concrete, significantly impacts the elastic modulus through its microstructure and mechanical properties. The ITZ typically exhibits higher porosity and a lower hydration product density compared to the bulk paste, resulting in an elastic modulus notably lower than that of the cement paste matrix. This creates a “short board” effect during stress transfer [18]. Some studies have shown that factors such as the thickness and pore distribution within the ITZ diminish the effective stiffness, consequently reducing the overall elastic modulus of the concrete [19].

Concrete is regarded as a three-phase composite material, of which the elastic modulus is closely related to the aggregate, matrix, and interfacial transition zone (ITZ). The volume fraction of the aggregate, porosity of the matrix, and the thickness and porosity of the ITZ are thus the key variables affecting the elastic modulus of concrete. The ITZ in concrete is the weak region between aggregates and bulk paste, with the thickness of approximately 20–50 μm. It is characterized by a high water–cement ratio, high porosity, and oriented arrangement of calcium hydroxide crystals, resulting in inferior mechanical properties and durability compared to the bulk paste [20,21,22]. Scholars have conducted extensive research on predictive models for the elastic modulus of ordinary concrete. To study the multiphase composition characteristics of concrete, a three-phase spherical model theory to predict the effective elastic modulus of concrete was developed [23]. Based on the three-phase model, a four-phase spherical model theory was proposed to further assess the influence of maximum aggregate particle size and ITZ on the elastic modulus of concrete [24]. The study revealed that both the ITZ layer and the maximum aggregate particle size significantly impact the elastic modulus of concrete. Enhancing the elastic modulus of the ITZ by incorporating silica fumes or reducing the thickness of the ITZ by lowering the water–binder ratio can effectively increase the elastic modulus of concrete. However, in their theoretical study, the overlap with the ITZ was ignored, resulting in higher predicted values and affecting the accuracy of the prediction. Gao et al. [25] proposed a numerical model involving the effect of ITZ overlapping, deriving a numerical approach for concrete’s compression properties. Unfortunately, this model also has limitations as it did not consider the non-uniform characteristics of the ITZ. Jia et al. [26] established a novel model for assessing the elastic modulus of fly ash concrete based on the three-phase sphere model, for purpose of investigating the influence mechanism of sustained loading on concrete. The results indicate that the elastic modulus after loading exhibits a minor increment compared to unloaded conditions. It is worth noting that the model did not consider the influence of different particle sizes of aggregates. Hashmi et al. [27] experimentally and theoretically studied the compressive strength and elastic modulus of fly ash concrete specimens at different ages. The results demonstrate that fly ash concrete exhibits higher growth rates in both compressive strength and elastic modulus compared to ordinary concrete, with the increase in curing time. In addition, this enhancement effect is most significant at a 30% fly ash replacement level. It is noteworthy that this analytical model only modified empirical formulas and failed to fully consider the impact of multiple factors. Wang et al. [28,29] used Brownian motion algorithm to evaluate the impact of the ITZ on ionic diffusion and found that the non-uniformity of the interface transition zone has a significant impact on the chloride ion transport performance of concrete, which cannot be ignored. Additionally, Tian et al. [30] employed finite element simulations to evaluate the impact of the ITZ on ionic diffusion. The computational results revealed that the diffusivity of the ITZ is approximately 40 times that of the bulk paste, and increases in either ITZ diffusivity or thickness will accelerate ionic migration in concrete. Chen et al. [31] developed a novel meshing technique by discretizing concrete models with an arbitrary ITZ thickness. The results demonstrated that the aggregate shape has negligible influence on the elastic modulus of concrete, whereas ITZ thickness is the critical parameter. Song et al. [32] established a three-phase finite element model which comprises a homogeneous bulk cement paste, aggregates, and ITZ. Based on finite element calculations, they assessed the ITZ’s influence on concrete’s macroscopic properties, finding that the elastic modulus of the ITZ exerts a greater impact on effective performance than ITZ thickness does. Although using grid subdivision was beneficial for model processing, it could lead to a significant increase in computer running time. Ren et al. [33] used the discrete element method to study the influence of the ITZ on the micro-mechanical properties of concrete. The results indicate that with the increase in ITZ strength, the compressive strength shows a non-linear increasing trend. This study has certain limitations as it did not consider the impact of fly ash on the mechanical properties of concrete. Liang et al. [34] studied the effects of recycled aggregate and ITZ properties on the elastic modulus of concrete using a recycled concrete aggregate model. Specifically, to simplify the calculation process, this study also did not consider the non-uniform characteristics of the ITZ. It is worth noting that the above theoretical models are all based on the assumption of ITZ uniformity, which has a certain gap with the non-uniform characteristics of the actual ITZ [35,36]. In addition, there is still a lack of research on the elastic modulus degradation mechanism of fly ash concrete in leached environments. Considering the issues in the aforementioned research, this study has three merits: Firstly, the overlap and non-uniformity of the ITZs will be considered in the developed model. Secondly, the influence of fly ash content on the calcium leaching resistance of concrete will be studied and analyzed. Thirdly, through sensitivity analysis, the influence of various factors on the elastic modulus of dissolved fly ash concrete will be fully explored.

By repeatedly using the two-phase composite sphere model, this study will establish a predictive model for the elastic modulus of leached fly ash concrete, comprehensively considering key parameters such as fly ash content, heterogeneous characteristics of the ITZ, and water-to-binder ratio. Through systematic experimental validation, the model’s reliability and predictive accuracy will be confirmed. Based on that method, this study will analyze the influence patterns of critical factors (including water-to-binder ratio, aggregate volume fraction, fly ash content, and interfacial transition zone thickness) on the elastic modulus of leached fly ash concrete. The findings will provide a significant theoretical basis for accurately evaluating the degradation mechanisms of mechanical properties and predicting the durability of fly ash concrete in leaching environments.

## 2. Experimental Study on Elastic Modulus of Dissolved Fly Ash Concrete

### 2.1. Materials

These experiments utilize P.O 42.5 ordinary Portland cement with densities of *ρ_c_ =* 3150 kg/m^3^ for cement and *ρ*_FA_ = 2300 kg/m^3^ for fly ash. Key chemical compositions are detailed in Table 1. River sand serves as a fine aggregate (density: 2650 kg/m^3^), while crushed stone is used as a coarse aggregate (apparent density: 2700 kg/m^3^). Aggregate gradation follows the Fuller distribution, with largest and smallest particle sizes of D_max_ = 16.0 mm and D_min_ = 0.3 mm, respectively. Tap water (pH: 6.8) is used for specimen mixing and curing. According to previous research, the typically adopted fly ash replacement ratio ranges from 10% to 30% by mass of binder materials [37]. However, it should be noted that specialized concrete formulations incorporating high-volume fly ash at 40–50% replacement levels have been developed [38], primarily aimed at reducing cement consumption and lowering carbon emissions. In this experiment and simulation, the mix proportion of fly ash concrete is based on conventional conditions, consequently establishing the fly ash replacement range at 0% to 20%. Fly ash replacement levels (FA) are thus set at 0%, 10%, and 20% with a constant water-to-binder ratio (w/b) of 0.5 and aggregate volume fraction (*f_a_*) of 45%. Mixture proportions for each concrete group are presented in Table 2.

### 2.2. Accelerated Calcium Leaching Test

**Figure 1:**
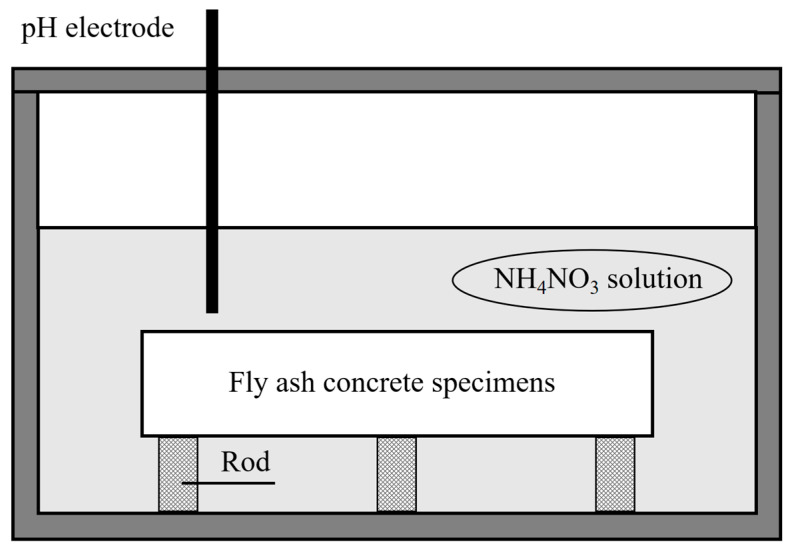
Self-designed experimental setup.

Under natural conditions, the leaching process is exceedingly slow. To accelerate that process, this experiment employed the chemical reagent acceleration method using NH_4_NO_3_ solution immersion [39]. First, fly ash concrete specimens are fabricated as cylinders measuring 100 mm × 500 mm. Additionally, seven specimens of the identical mix proportion are prepared: three designated for leaching depth measurement, three for elastic modulus testing, and one for mercury intrusion porosimetry (MIP) testing. After casting, the specimens are kept stewing for 24 h before demolding, and then subsequently placed in a standard curing room for 28 days. Following curing, a dual-blade numerical control v-strop cutter is used to section the specimens into 200 mm tall cylindrical test specimens. To ensure leaching occurred exclusively on the lateral surfaces, the upper and lower ends of the specimens are sealed with wax. The specimens are then immersed in a 6 mol/L NH_4_NO_3_ solution; the experimental setup is illustrated in Figure 1. Crucially, the pH value of the solution is monitored daily and replenished promptly to maintain it within the range of 8.5–9.2, thereby ensuring effective leaching.

**Figure 2:**
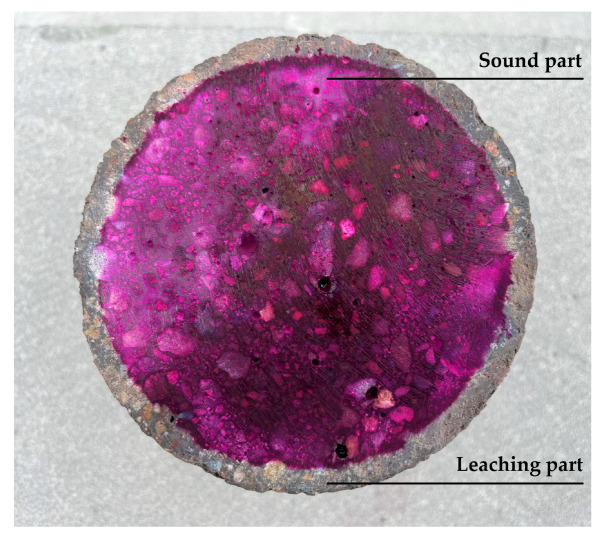
Acid–base indication experiment at leaching time of 14 d.

At specified leaching durations (3, 14, 28, 45, and 90 d), specimens are removed from the container and air-dried. Before testing, a handheld cutter is used to perform the mid-height sectioning to expose the evaluation surface. The colorimetric method involved spraying phenolphthalein reagent onto the leached concrete surface. Regions unaffected by leaching exhibited a purple–red color due to alkalinity, while leached areas remained unchanged. The colorimetric test results for the specimen leached for 14 days are shown in Figure 2. Sixteen points are uniformly selected along the concrete leaching front. The digital vernier caliper is used to measure the distance from the specimen edge to the color boundary line at each point. The leaching depth was determined via weighted averaging of these measurements. Specimens of the same mix proportion and leaching duration are subjected to elastic modulus testing using a hydraulic servo universal testing machine.

### 2.3. Material Characterization of Mercury Intrusion Porosimetry (MIP)

**Figure 3:**
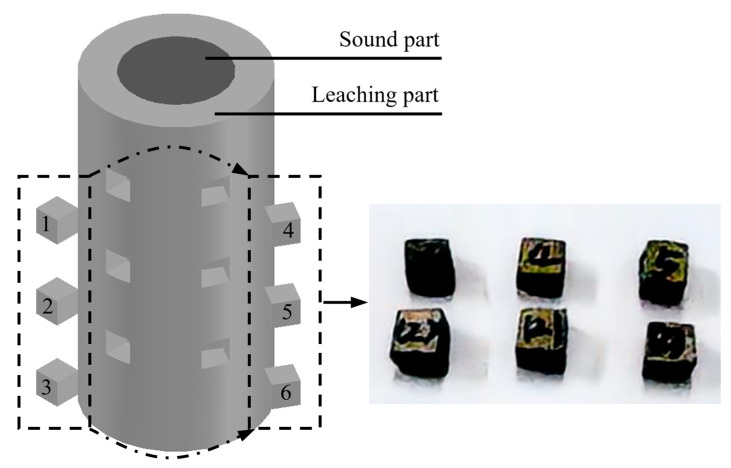
Sampling process for MIP testing.

The widely established techniques for pore structure analysis in porous media include Brunauer–Emmett–Teller (BET) gas adsorption and mercury intrusion porosimetry (MIP). The BET method quantifies the specific surface area and pore architecture through gas adsorption isotherms, recognized as a robust and validated characterization approach. Mercury intrusion porosimetry operates on the principle of pressurized mercury intrusion/extrusion, where pore parameters are derived from the equilibrium between applied pressure and mercury surface tension. It should be noted that this study exclusively employs MIP. For cement-based materials, MIP testing mainly consists of three steps:

(1) Specimens are cut into 10 ± 2 mm cubes and dried at 105 °C for 24 h until achieving a constant mass (<0.1% variation). The sampling process can be seen in Figure 3, and six samples are selected from each specimen. After vacuum degassing (<10 Pa for 30 min), the geometric volume is measured with 0.1 mm precision. This stage eliminates moisture and entrapped air in accessible pores.
(2) Mercury intrusion testing following ISO 15901-1 [40]: the test starts at 0.1 psi (detecting >360 μm pores) and progresses to 60,000 psi (3.6 nm pores). Each pressure step maintains 120 s equilibration, with 0.1 μL resolution capacitance measurements. Critical 10–100 μm pores are analyzed using 0.5 psi increments between 20 and 200 psi.
(3) Total porosity = (intruded Hg volume/geometric volume) × 100%.

### 2.4. Material Characterization of Scanning Electron Microscope (SEM)

In this experiment, an SEM is used to detect the changes in porosity of fly ash concrete before and after calcium leaching. The testing process mainly includes two steps:

(1) Sample preparation: Cut concrete specimens (10 mm × 10 mm × 5 mm) from representative areas, sequentially polish with 400–1500-grit abrasive papers, then coat with 10–15 nm silver film via ion sputtering. Dry at 60 °C and store airtight to prevent hydration interference, ensuring surface roughness < 1 μm.
(2) SEM analysis: Place samples in SEM chamber (5 × 10^−3^ Pa vacuum), and then set accelerating voltage (15–30 kV) and working distance (8–10 mm). Acquire images via SE/BSE modes coupled with EDS elemental mapping. Initially scan at 500× to locate features, then switch to 2000–5000× for hydrate morphology and pore structure observation.

### 2.5. Results and Discussion

According to the above experimental method, the leaching depth *l*, leaching degree D_C_, and elastic modulus E_fc_ of three groups of fly ash concrete specimens with different leaching times A0, A1, and A2 are measured. The leaching degree of the concrete specimens can be calculated as follows:

(1) $$D_{c}=\frac{S_{1}}{S_{0}}=\frac{S_{0}-S_{2}}{S_{0}}$$

where S_0_, S_1_, and S_2_ represent the total cross-sectional area, leached cross-sectional area, and undissolved cross-sectional area of the specimen, respectively.

The changes in leaching depth, degree, and elastic modulus of each group of specimens with leaching time are shown in Table 3.

Based on the tabulated data, it is observed that at a constant water–binder ratio, the elastic modulus of fly ash-incorporated concrete exhibits a significantly lower rate of decline compared to ordinary concrete as the leaching degree increases. This indicates that incorporating an appropriate amount of fly ash can effectively enhance the resistance to dissolution leaching. This phenomenon may be attributed to the secondary hydration reaction of fly ash: the active SiO_2_ and Al_2_O_3_ in fly ash react with calcium hydroxide (CH) generated from cement hydration to form a dense calcium silicate hydrate (C-S-H) gel, which effectively reduces porosity and simultaneously blocks dissolution pathways. In contrast, for ordinary concrete, after the preferential leaching of the hydration product CH, the pore connectivity sharply increases, resulting in an accelerated deterioration of the elastic modulus.

**Figure 4:**
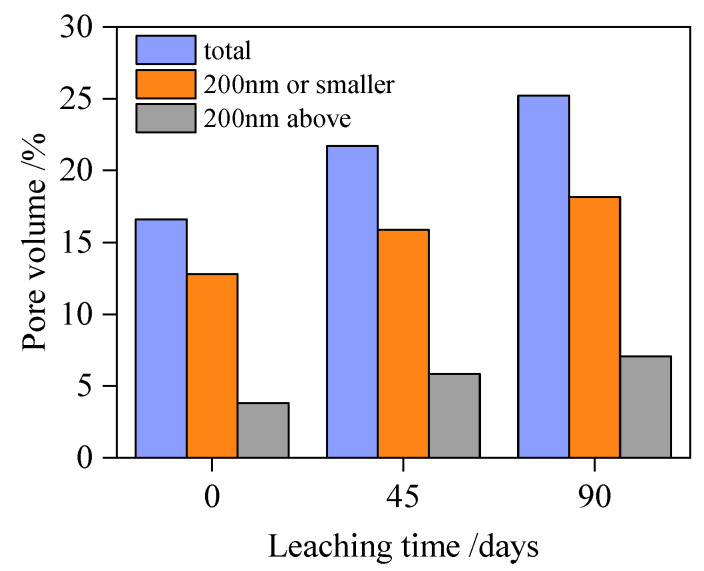
The change in porosity of leached fly ash concrete.

The evolution of porosity in fly ash concrete specimen A2 under the leaching effect is measured using MIP testing, as shown in Figure 4, wherein after leaching, the number of pores larger than 200 nm and smaller than 200 nm within the specimen increases. Specifically, the dissolution of calcium hydroxide (CH) generates larger pores (typically >200 nm), while the decalcification of C-S-H gel produces smaller pores (generally <200 nm).

**Figure 5:**
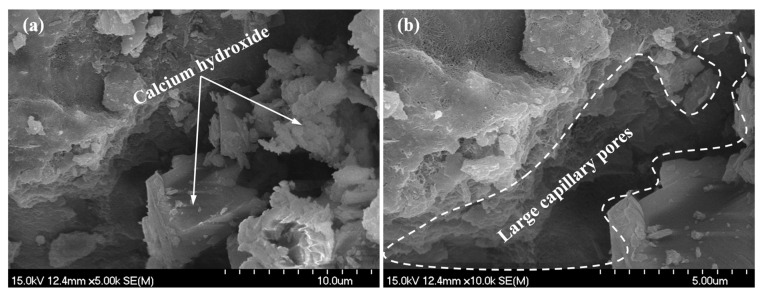
SEM detection for matrix: (**a**) before leaching; (**b**) after leaching.

**Figure 6:**
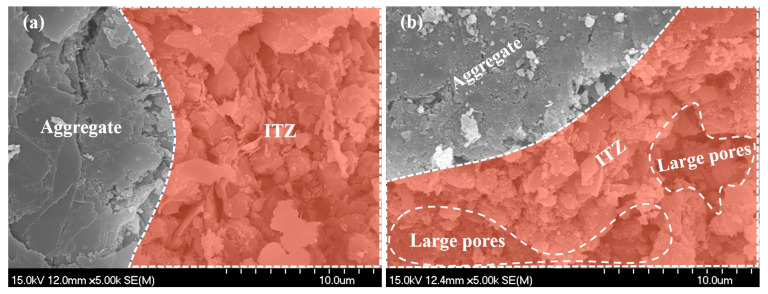
SEM detection for ITZ: (**a**) before leaching; (**b**) after leaching.

**Figure 7:**
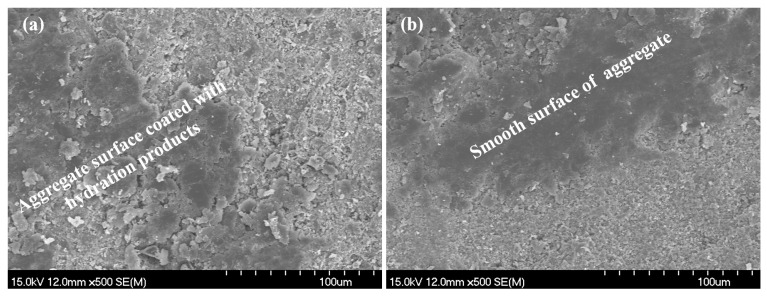
SEM detection for aggregate surface: (**a**) before leaching; (**b**) after leaching.

For specimen A2, the results of SEM testing are shown in Figure 5, Figure 6 and Figure 7. Figure 5a and Figure 5b, respectively, show the hydration products and pore distribution before and after leaching in the matrix. The figures show that after leaching, there is a phenomenon of calcium hydroxide dissolution and a significant increase in porosity in the microstructure of the bulk paste. Figure 6a,b display the microstructure morphology of the ITZ before and after leaching, whose variation pattern is consistent with the case in the matrix. As Figure 7 shows, the surface of the leached aggregate becomes smooth, attributed to the hydration products accumulated on the surface of the aggregate being dissolved.

## 3. Prediction Model for Elastic Modulus of Leached Fly Ash Concrete

Building upon the experimental findings (i.e., accelerated leaching test, MIP, and SEM) of leached fly ash concrete with 0–20% FA replacement, the change characteristics of the elastic modulus, porosity, and microstructure can be analyzed. These experimental results can provide a theoretical basis for subsequent theoretical modeling and data support for model validation.

### 3.1. Prediction Theory of Elastic Modulus of Two-Phase Composite Materials

**Figure 8:**
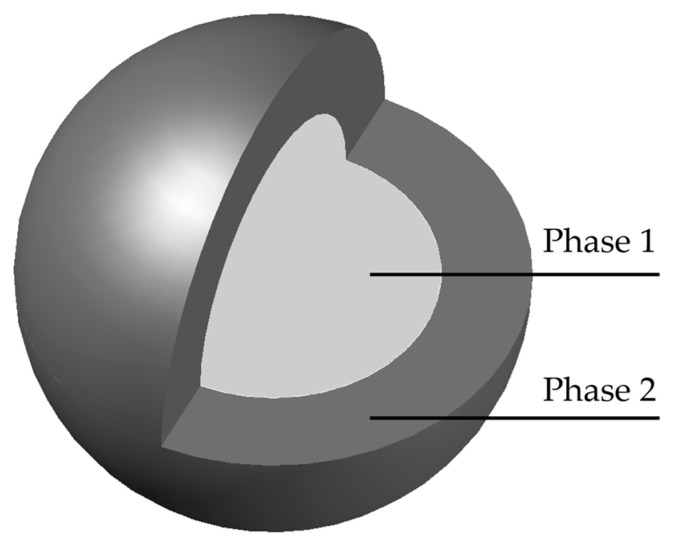
Two-phase composite sphere model.

At the macro level, the composition of the two-phase composite sphere consists of two major parts: the inclusion phase and the matrix phase, as shown in Figure 8. This section applies homogenization theory to calculate two-phase composite materials as equivalent spherical elements with an identical elastic modulus. It is noteworthy that the two-phase composite sphere theory establishes an effective analytical framework for probing composite material properties. This model assumes that the composite comprises numerous periodically distributed two-phase spherical units, each consisting of a core and a shell structure. The core represents the inclusion phase, while the shell corresponds to the matrix phase. Both core and shell materials are treated as isotropic and homogeneous, without accounting for microstructural orientation or spatial heterogeneity in material properties [41].

Macroscopically, homogenization methods enable the simulation of composites as equivalent media with effective elastic moduli. Using elasticity theory, the effective bulk modulus K_e_ and shear modulus G_e_ of the two-phase composite spheres depicted in Figure 8 can be determined by [41]

(2) $$K_{e}=K_{2}+\frac{(K_{1}-K_{2})f_{1}}{1+[(1-f_{1})(K_{1}-K_{2})/(K_{2}+4G_{2}/3)]}$$

where K_1_ and K_2_ represent the effective bulk moduli of phases 1 and 2; G_1_ and G_2_ represent the effective shear moduli of phases 1 and 2; and f_1_ is the volume fraction of phase 1.

(3) $$G_{e}=G_{2}\frac{\omega_{2}\sqrt{{\omega_{2}}^{2}-4\omega_{1}\omega_{3}}}{2\omega_{1}}$$

(4) $$\begin{array}{ll} \omega_{1}= & 8(G_{1}/G_{2}-1)(4-5\nu_{2})u_{1}f_{1}^{10/3}-2[63(G_{1}/G_{2}-1)u_{2}+2u_{1}u_{3}]f_{1}^{7/3}\\ & +252(G_{1}/G_{2}-1)u_{2}f_{1}^{5/3}-50(G_{1}/G_{2}-1)(7-12\nu_{2}+8\nu_{2}^{2})u_{2}f_{1}\\ & +4(7-10\nu_{2})u_{2}u_{3} \end{array}$$

(5) $$\begin{array}{ll} \omega_{2}= & -4(G_{1}/G_{2}-1)(1-5\nu_{2})u_{1}f_{1}^{10/3}+4[63(G_{1}/G_{2}-1)u_{2}+2u_{1}u_{3}]f_{1}^{7/3}\\ & -504(G_{1}/G_{2}-1)u_{2}f_{1}^{5/3}+150(G_{1}/G_{2}-1)(3-\nu_{2})\nu_{2}u_{2}f_{1}\\ & +3(15\nu_{2}-7)u_{2}u_{3} \end{array}$$

(6) $$\begin{array}{ll} \omega_{3}= & 4(G_{1}/G_{2}-1)(5\nu_{2}-7)u_{1}f_{1}^{10/3}-2[63(G_{1}/G_{2}-1)u_{2}+2u_{1}u_{3}]f_{1}^{7/3}\\ & +252(G_{1}/G_{2}-1)u_{2}f_{1}^{5/3}+25(G_{1}/G_{2}-1)(\nu_{2}^{2}-7)u_{2}f_{1}\\ & -(5\nu_{2}+7)u_{2}u_{3} \end{array}$$

(7) $$u_{1}=(G_{1}/G_{2}-1)(49-50\nu_{1}\nu_{2})+35(G_{1}/G_{2})(\nu_{1}-2\nu_{2})+35(2\nu_{1}-\nu_{2})$$

(8) $$u_{2}=5\nu_{1}(G_{1}/G_{2}-4)+7(G_{1}/G_{2}+4)$$

(9) $$u_{3}=(G_{1}/G_{2})(8-10\nu_{2})+(7-5\nu_{2})$$

where v_1_ and v_2_ denote the Poisson’s ratios of phase 1 and phase 2; u_1_, u_2_, and u_3_ denote the functions of parameters G_1_, G_2_, v_1_, and v_2_; and ω_1_, ω_2_, and ω_3_ denote the functions of parameters (G_1_, G_2_, v_1_, and v_2_), volume fraction of phase 1 (f_1_), and functions (u_1_, u_2_, and u_3_).

Once G_e_ and K_e_ are known, E_e_ is solved by

(10) $$E_{e}=\frac{3}{1/G_{e}+1/3K_{e}}$$

where E_e_ represents the effective elastic modulus.

### 3.2. Inhomogeneous Model of ITZ

**Figure 9:**
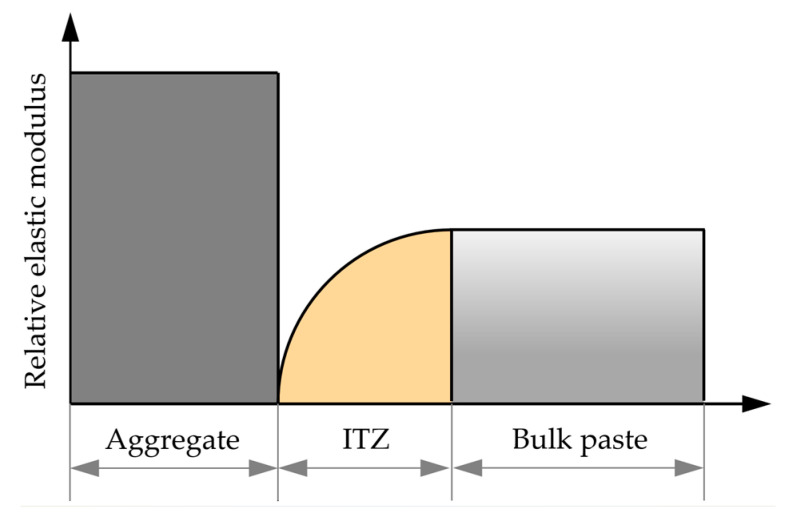
Relative elastic modulus of each component of fly ash concrete.

**Figure 10:**
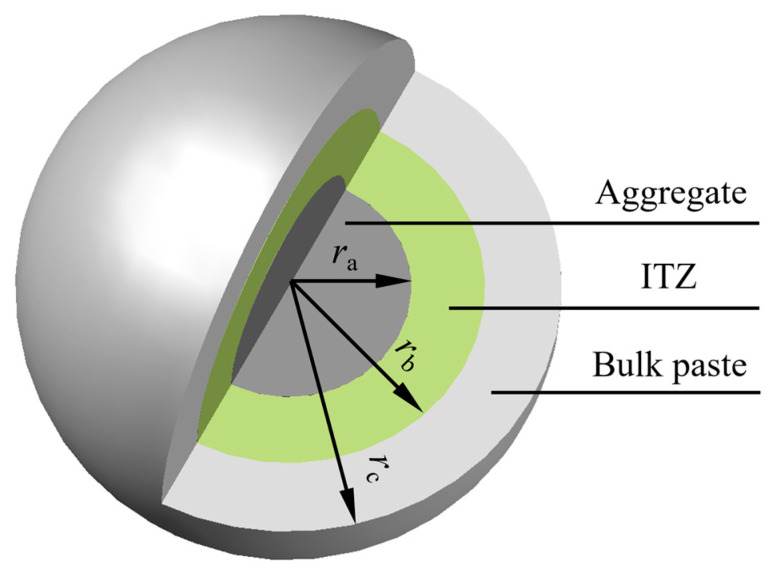
Three-phase composite sphere model of fly ash concrete.

From the mesoscopic perspective, concrete is a three-phase composite material composed of cement bulk paste, ITZ, and aggregate [42,43]. The relative magnitudes of the elastic moduli for the various components of concrete are illustrated in Figure 9 [44]. This section adopts the concentric spheres model shown in Figure 10 to represent the three-phase fly ash concrete. Within this model, the aggregate has an equivalent radius of r_a_; the region between the inner radius r_a_ and the outer radius r_b_ represents the ITZ; and the region between the inner radius r_b_ and the outer radius r_c_ represents the fly ash–cement paste.

To determine the volume fraction of the interface f_i_ and bulk cementitious paste f_bulk_, it is first necessary to analyze the size distribution of the aggregate. For Fuller grading, the cumulative distribution function of aggregate particles with respect to particle size can be expressed as

(11) $$P(D)=\frac{nD_{min}^{n}D_{max}^{n}}{(D_{max}^{n}-D_{min}^{n})D^{n+1}}$$

where n is the grading type coefficient of the aggregate. For the Fuller distribution of the aggregate, n is fixed at 2.5.

The f_i_ can be expressed as the function of the ITZ thickness h, which can be expressed as [45]

(12) $$f_{i}=(1-f_{a})\text{\{}1-\exp[-\pi \rho (t_{1}h+t_{2}h^{2}+t_{3}h^{3})]\text{\}}$$

(13) $$t_{1}=\frac{4\langle R^{2}\rangle }{1-f_{a}}$$

(14) $$t_{2}=\frac{4\langle R\rangle }{1-f_{a}}+\frac{8\pi \rho {\langle R^{2}\rangle }^{2}}{{\left(1-f_{a}\right)}^{2}}$$

(15) $$t_{3}=\frac{4}{3(1-f_{a})}+\frac{16\pi \rho \langle R\rangle {\langle R^{2}\rangle }^{2}}{3{\left(1-f_{a}\right)}^{2}}+\frac{64\lambda \pi^{2}\rho^{2}{\langle R^{2}\rangle }^{3}}{27{\left(1-f_{a}\right)}^{3}}$$

where *ρ* is the number of aggregate particles per unit volume; <R> is the average radius of the aggregate; and <R^2^> is the average mean square radius of the aggregate.

The volume of bulk paste f_bulk_ can thus be expressed as

(16) $$f_{bulk}=1-f_{i}-f_{a}$$

Due to the edge wall effect on the surface of aggregates, the distribution of cementitious particles (cement and fly ash) in the interface transition zone exhibits a gradient distribution. According to Crumbie’s research [46], the initial distribution density of cementitious materials can be expressed as

(17) $$f_{b}(r)=\left\{\begin{array}{l} f_{b,bulk}\sum_{j=1}^{4}(b_{j}/b_{0}){\left(\frac{r-r_{a}}{r_{b}-r_{a}}\right)}^{j},r_{a}\leq r\leq r_{b}\\ f_{b,bulk},r_{b}\leq r\leq r_{c} \end{array}\right.$$

where r is the distance from the ITZ or bulk paste to the center of the aggregate; and b_0_ = b_1_ + b_2_ + b_3_ + b_4_. The relationship between b_j_ (j = 1, 2, 3, and 4) and the initial water–cement ratio (w/b)_0_ is expressed as follows:

(18) $$b_{1}=4.670-5.228{\left(w/b\right)}_{0}$$

(19) $$b_{2}=-10.569+12.700{\left(w/b\right)}_{0}$$

(20) $$b_{3}=9.950-12.195{\left(w/b\right)}_{0}$$

(21) $$b_{4}=-3.397+4.195{\left(w/b\right)}_{0}$$

**Figure 11:**
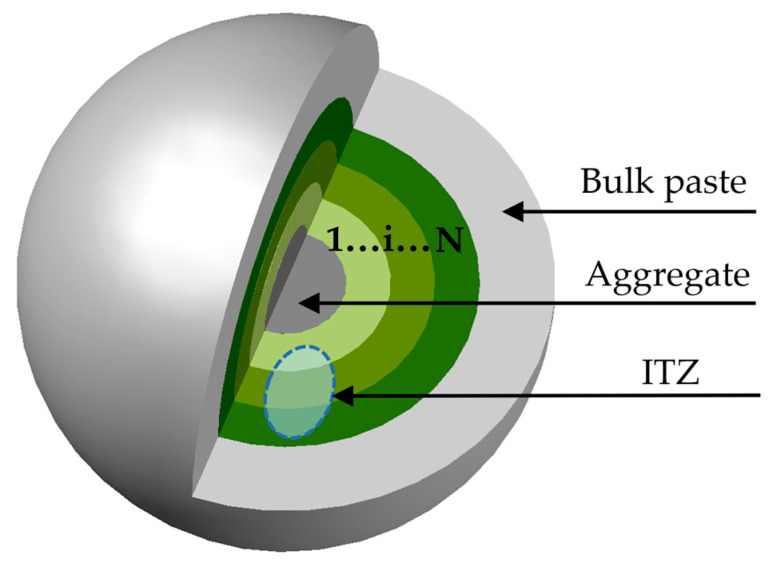
Non-uniformity of ITZ divided into N layers.

Considering the non-uniformity of the ITZ, it is necessary to divide it into *N* concentric shell elements of equal thickness, as shown in Figure 11, to calculate the elastic modulus and shear modulus of each shell element using the two-phase composite sphere theory. Since the volume of cementitious material in the composite ball is equal to the actual volume of cementitious material in fly ash concrete, f_b,bulk_ can be calculated as follows:

(22) $$f_{b,bulk}=\frac{\left(r_{c}^{3}-r_{a}^{3}\right)\left[\rho_{c}\mathrm{FA}+\rho_{\mathrm{FA}}\left(1-FA\right)\right]}{\left[\rho_{c}\mathrm{FA}+\rho_{\mathrm{FA}}\left(1-FA\right)+\rho_{c}\rho_{\mathrm{FA}}{\left(w/b\right)}_{0}\right]\left[\left(r_{c}^{3}-r_{a}^{3}\right)+6\sum_{j=1}^{4}\sum_{k=1}^{3}\frac{b_{j}r_{a}^{\left(3-k\right)}{\left(r_{b}-r_{a}\right)}^{k}}{b_{0}\left(j+k\right)\left(k-1\right)!\left(3-k\right)!}\right]}$$

The local water–cement ratio (w/b)_r_ at any point in the ITZ region can be calculated as follows:

(23) $${\left(w/b\right)}_{r}=\frac{1-f_{b}(r)}{f_{b}(r)\rho_{FA}\rho_{c}/\left[\rho_{c}FA+(1-FA)\rho_{FA}\right]}$$

### 3.3. Porosity Calculation After Calcium Leaching

In the above ITZ uniformly divided into N layers, according to Powers’ model [47,48], the capillary pores of any layer of bulk paste can be expressed as follows:

(24) $$\varphi_{cap}^{0}(r)=\frac{m_{w}/\rho_{w}-0.36a(m_{c}+0.30m_{FA})/\rho_{W}}{m_{c}/\rho_{c}+m_{w}/\rho_{w}+m_{FA}/\rho_{FA}}$$

(25) $$\alpha =\alpha_{ult}\cdot exp\left(-{\left(\frac{\tau }{t}\right)}^{\theta }\right)$$

(26) $$\alpha_{ult}=\frac{1.031{\left(w/b\right)}_{r}}{0.194+{\left(w/b\right)}_{r}}+exp(-0.297-9.73P_{C_{4}AF}\cdot P_{cem}-325P_{Na_{2}Oeq}\cdot P_{cem}-8.9P_{FA}\cdot P_{\mathrm{FA-CaO}})$$

(27) $$Na_{2}O_{eq}=0.658K_{2}O+Na_{2}O$$

(28) $$\tau =exp\left(2.95-0.972P_{C_{3}S}\cdot P_{cem}+152P_{Na_{2}O}\cdot P_{cem}+4.0P_{FA}\cdot P_{\mathrm{FA-CaO}}\right)$$

(29) $$\theta =\exp\left(-0.418+2.66P_{C_{3}A}\cdot P_{\mathrm{cem}}\right)$$

where α represents the hydration degree; m_c_, m_w_, and m_FA_ represent the mass of cement, water, and fly ash in the mixing proportion, respectively; α_ult_ is the ultimate degree of hydration; t is the curing time; τ and θ represent parameters related to the chemical composition of cement and fly ash; P_C4AF_, P_Na2Oeq_, P_C3S_, P_C3A_, and P_Na2O_ denote the mass ratios of C_4_AF, Na_2_Oeq, C_3_S, C_3_A, and Na_2_O to the cement mass, respectively; P_cem_ and P_FA_ are the mass ratios of cement and fly ash to the total binder mass, respectively; and P_FA-CaO_ is the mass ratio of CaO to fly ash mass in fly ash.

The decalcification of C-S-H and CH after calcium leaching will lead to additional porosity, thereby reducing the elastic modulus of fly ash concrete. To quantify this impact, it is necessary to clarify the content of CH and C-S-H generated during cement hydration and secondary reaction of fly ash. According to existing research [49,50], the hydration reactions of cement and fly ash can be represented as follows:

(30) $$C_{3}S+5.3H=1.3\mathrm{CH}+C_{1.7}{\mathrm{SH}}_{4}$$

(31) $$C_{2}S+4.3H=0.3\mathrm{CH}+C_{1.7}{\mathrm{SH}}_{4}$$

(32) $$\mathrm{AS}\cdot \mathrm{xS}\cdot \mathrm{yC}+(2+1.1x-y)\mathrm{CH}+(6+2.8x+{y)H=\mathrm{xC}}_{1.1}{\mathrm{SH}}_{3.9}+C_{2}{\mathrm{ASH}}_{8}$$

(33) $$\begin{array}{c} m_{\mathrm{CH}}=(1.32P_{\mathrm{CaO}}-1.85P_{{\mathrm{SiO}}_{2}}-2.91P_{{\mathrm{Al}}_{2}O_{3}}-0.93P_{{\mathrm{Fe}}_{2}O_{3}})m_{c}\alpha \\ -1.44(1.517P_{{\mathrm{FA-SiO}}_{2}}+2.386P_{{\mathrm{FA-Al}}_{2}O_{3}})m_{\mathrm{FA}}\alpha \end{array}$$

where H, S, and C denote H_2_O, SiO_2_, and CaO, respectively; C_1.7_SH_4_ and C_1.1_SH_3.9_ represent the cement hydration product and pozzolanic reaction product; AS denotes Al_2_O_3_ + SiO_2_; x and y are parameters related to the chemical composition of fly ash; mCH denotes the mass of calcium hydroxide; P_CaO_, P_SiO2_, P_Al2O3_, and P_Fe2O3_ are the mass ratios of CaO, SiO_2_, Al_2_O_3_, and Fe_2_O_3_ to cement mass, respectively; and P_FA-SiO2_ and P_FA-Al2O3_ are the mass ratios of SiO_2_ and Al_2_O_3_ to fly ash mass.

The increase in the porosity of the ITZ and bulk paste caused by C-S-H and CH decalcification can therefore be calculated as follows [51,52]:

(34) $$\varphi_{\mathrm{ITZ}}=f_{i}(m_{\mathrm{CH-ITZ}}/\rho_{\mathrm{CH}}+0.647m_{C_{1.7}{\mathrm{SH}}_{4}\text{-ITZ}}/\rho_{C_{1.7}{\mathrm{SH}}_{4}}+0.407m_{C_{1.1}{\mathrm{SH}}_{3.9}\text{-ITZ}}/\rho_{C_{1.1}{\mathrm{SH}}_{3.9}})D_{c}$$

(35) $$\varphi_{\mathrm{bulk}}=f_{\mathrm{bulk}}(m_{\mathrm{CH-bulk}}/\rho_{\mathrm{CH}}+0.647m_{C_{1.7}{\mathrm{SH}}_{4}\text{-bulk}}/\rho_{C_{1.7}{\mathrm{SH}}_{4}}+0.407m_{C_{1.1}{\mathrm{SH}}_{3.9}\text{bulk}}/\rho_{C_{1.1}{\mathrm{SH}}_{3.9}})D_{c}$$

where m_CH-ITZ_ and m_CH-bulk_ represent the CH mass generated by cement hydration in the ITZ and bulk paste, respectively; m_C1.7SH4-ITZ_ and m_C1.7SH4_ represent the mass of hydrated calcium silicate generated by cement hydration in the ITZ and bulk paste, respectively; m_C1.1SH3.9-ITZ_ and m_C1.1SH3.9-bulk_ represent the mass of hydrated calcium silicate generated by the reaction of fly ash in the ITZ and bulk paste, respectively; and *ρ*_C1.7SH4_, *ρ*_C1.1SH3.9_, and *ρ*_CH_ are the corresponding densities.

**Figure 12:**
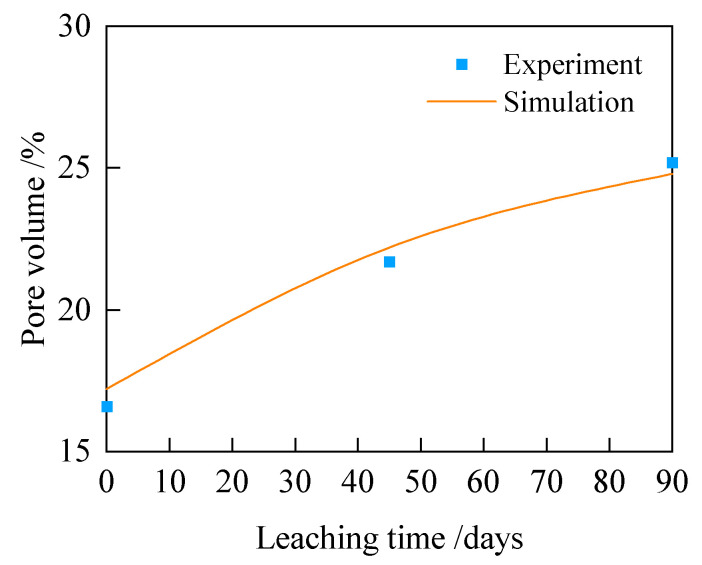
Comparison between calculated and experimental values of porosity of leached fly ash concrete.

To validate the effectiveness of the porosity calculation model for leached fly ash concrete established in this study, the calculated results are compared with the MIP test results. The comparison between the two is shown in Figure 12, wherein the experimental values are highly consistent with the calculated values, with a correlation coefficient of 0.971, thereby validating the effectiveness of the porosity calculation method.

### 3.4. Prediction for Elastic Modulus of Leached Fly Ash Concrete

Regarding the proposed heterogeneous ITZ, the porosity can be assumed to be equal at the ITZ–matrix interface. Due to the wall effect adjacent to the aggregate surface, the porosity at the aggregate–ITZ boundary (i.e., the aggregate surface) approaches unity. Due to the radial variation in porosity in the ITZ, the region of the ITZ is discretized into N equally thick spherical shell elements when calculating the elastic modulus of leached fly ash concrete. Based on the aforementioned chemical reaction equation, the porosity at different leaching degrees can be determined. When treating pores as the inclusion phase and solid as the matrix phase, the bulk modulus and shear modulus of each leached ITZ and bulk paste are computed using the two-phase composite sphere formulation. The elastic modulus of leached fly ash concrete is then calculated via a sequential homogenization method:

(1) Construct the initial composite sphere model: The aggregate and the primary ITZ shell unit constitute the first composite sphere, wherein the aggregate is treated as the inclusion phase and the primary interfacial shell as the matrix phase. Via the two-phase composite sphere formulation method, the effective bulk modulus and shear modulus of this composite sphere are calculated using the bulk moduli, shear moduli, and volume fractions of both the aggregate and primary interfacial shell.
(2) Construct the 2nd to Nth composite spheres sequentially: The first composite sphere and secondary ITZ shell unit are combined to form the second composite sphere, wherein the first composite sphere serves as the inclusion phase and the secondary interfacial shell as the matrix phase. Following an identical computational procedure, the bulk modulus and shear modulus of the k-th composite sphere (k = 2, 3, ..., N) are calculated using the two-phase composite sphere formulation. This process is iterated by successively combining the preceding composite sphere with the next ITZ shell unit. After N − 1 computational steps, the effective bulk and shear moduli for composite spheres 2 through N are obtained.
(3) Construct the (N + 1)-th composite sphere and derive the elastic modulus of leached concrete: Ultimately, the N-th composite sphere and cement paste matrix constitute the (N + 1)-th composite sphere, with the N-th composite sphere serving as the inclusion phase and the cement paste as the matrix phase. The bulk modulus K^(N+1)^ and shear modulus G^(N+1)^ of this composite sphere are calculated. Subsequently, the elastic modulus of leached concrete is obtained through the mathematical relationship between elastic modulus and the computed bulk/shear moduli.

**Figure 13:**
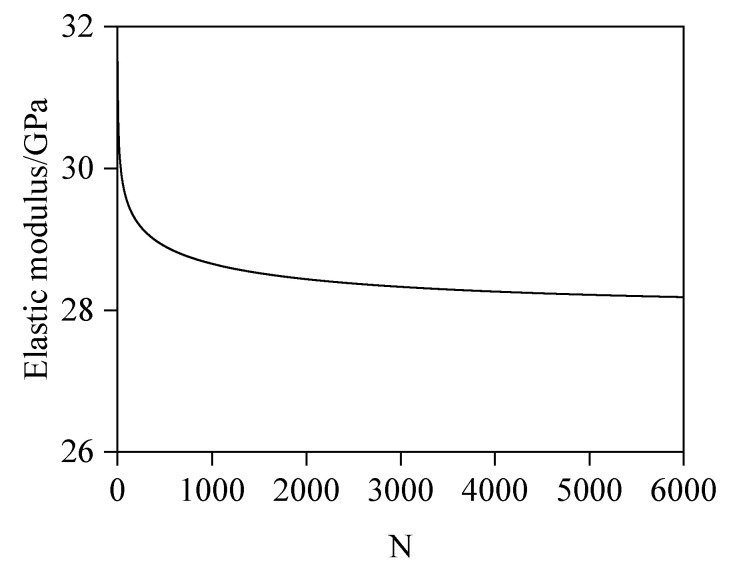
Variation in E with N.

**Figure 14:**
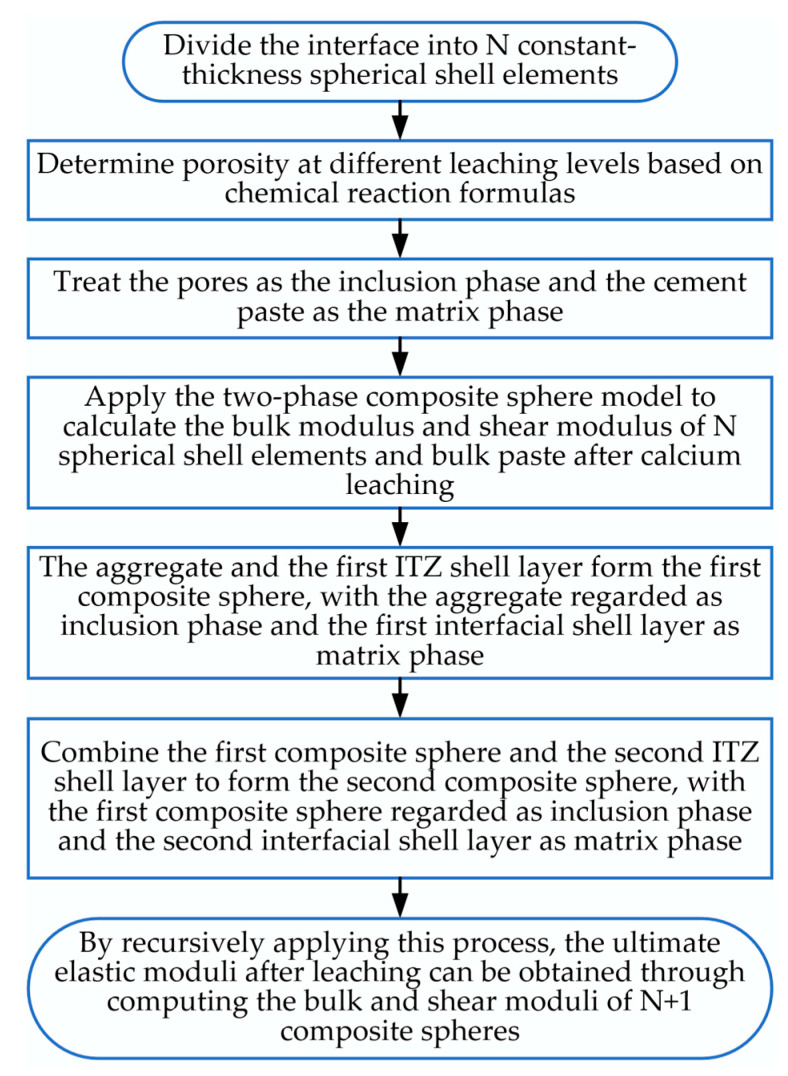
Calculation flowchart of elastic modulus of fly ash concrete.

Through analysis of computational results under varying N-values, this study establishes the variation pattern of elastic modulus with the number of spherical shell elements (N), as depicted in Figure 13. Significant solution oscillations occur at lower N-values, attributable to insufficient discretization refinement for accurately capturing radial variations in ITZ porosity, thereby compromising modulus calculation accuracy. As N increases, results progressively stabilize. Computational solutions are considered converged when N ≥ 3000, satisfying prescribed accuracy requirements. This convergence confirms that the discretized spherical shell elements sufficiently resolve interfacial porosity gradients, yielding reliable elastic modulus predictions for leached concrete. The complete computational workflow is illustrated in Figure 14.

## 4. Validation and Parametric Analysis

### 4.1. Model Validation

**Figure 15:**
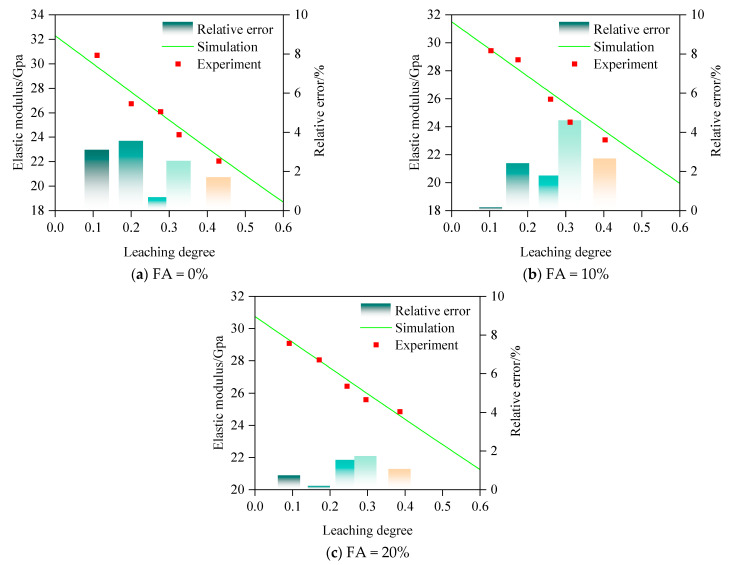
Experimental validation of prediction model.

To validate the effectiveness of the prediction method for the elastic modulus of fly ash concrete, the predicted values are compared with self-conducted experimental values derived from Section 2, and the mixture proportions of concrete are also detailed. The CTS-H600 hydraulic servo universal testing machine is employed to measure the elastic modulus of concrete specimens after leaching. In the corresponding theoretical calculations, the variables in the simulation calculation are consistent with the experimental variables. The morphology of the aggregate is assumed to be spherical. The range of aggregate particle size is 0.3–16 mm and follows the Fuller distribution. Some specific fixed parameters are assigned as follows, primarily referencing [53]: The bulk modulus and shear modulus of cement paste are fixed at 22.83 GPa and 11.77 GPa, respectively, with a Poisson’s ratio of 0.28. The bulk modulus and shear modulus of the aggregate are 43.79 GPa and 37.05 GPa, respectively, with a Poisson’s ratio of 0.17. The ITZ thickness is determined as 30 μm. As shown in Figure 15, the simulation results closely align with the experimental data, with correlation coefficients of 0.973, 0.975, and 0.910, respectively, validating the reliability of the predictive method. By calculating the relative error between the experimental and simulated values, as shown in Figure 15, the maximum relative error between the two is only 4.6%, further proving the effectiveness and rationality of the simulation method proposed in this study.

**Figure 16:**
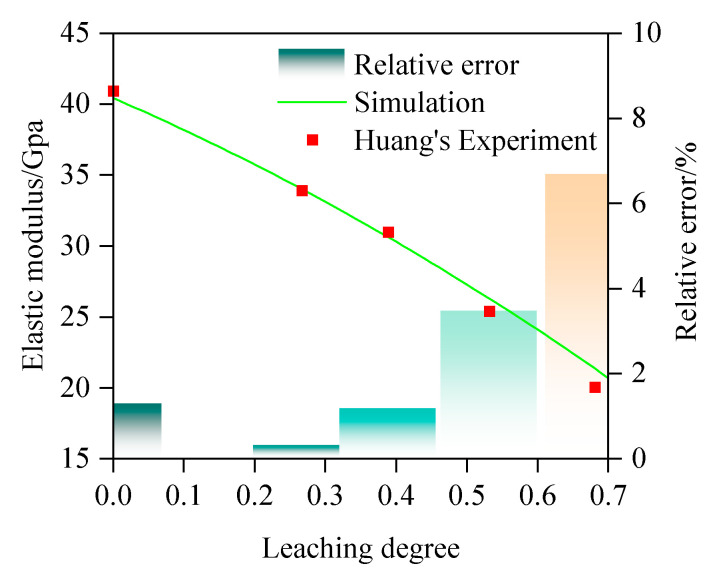
Experimental validation of prediction model.

To verify the general applicability of the established model, we also selected third-party experimental results for validation. The test results from Huang et al. [54] are selected for validation. In their experiments, they employed Type I ordinary Portland cement (ASTM C150 Grade 42.5) with a water-to-cement ratio of 0.53. The concrete mix design incorporated continuously graded aggregates (0.15–20.0 mm) adhering to Fuller’s optimal curve, maintaining a 70% aggregate volume fraction. Specimens were demolded 24 h post-casting and subsequently cured under standard conditions (20 ± 2 °C, >95% RH) for 28 days. Following curing, samples underwent chemical leaching treatment via immersion in 6 mol/L ammonium nitrate solution. The elastic modulus of leached specimens was determined using the MTS810 hydraulic servo universal testing machine (MTS Industrial Systems (China) Co., Ltd., Beijing, China). The specific fixed parameters (e.g., the bulk modulus and shear modulus of the aggregate, the morphology of the aggregate, etc.) used in the simulation are the same as those in the first validation case. The comparison between the simulation and experimental results is illustrated in Figure 16, showing that the simulation results are in good agreement with the experimental data. The corresponding correlation coefficient is calculated as 0.988. This comparison result further validates the reliability of the methodology proposed in this study. The relative error between the simulated values and Huang’s experimental values is shown in Figure 16, where the relative error of both is within 8%, which once again verifies the reliability of the simulation method employed in this study.

### 4.2. Parametric Analysis

#### 4.2.1. The Influence of w/b on E

**Figure 17:**
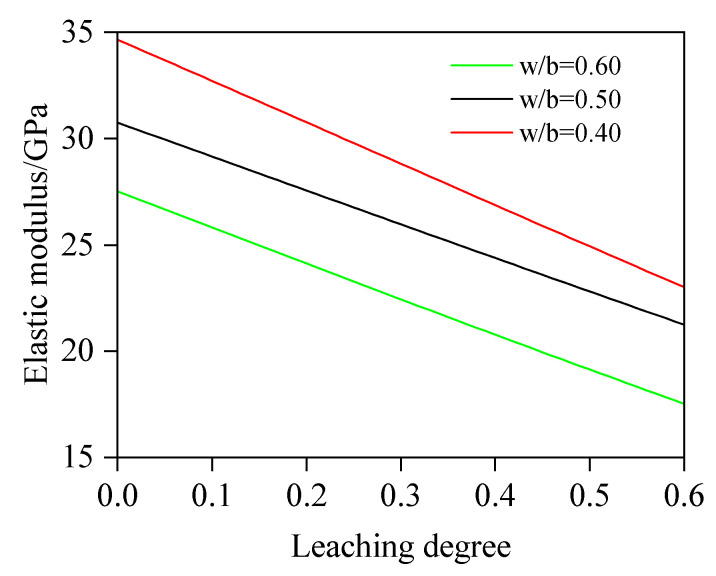
Effect of w/b on E.

In the simulation analysis, the parameter values are shown in Table 4. It is noteworthy that the morphology of the aggregate is assumed to be spherical. The range of aggregate particle size is 0.3–16 mm and follows the Fuller distribution, and the aggregate adopts Fuller gradation. The relationship between E and D_c_ at different w/b values is shown in Figure 17, demonstrating that under identical D_c_ values, E decreases with an increasing w/b, primarily due to prominently elevated porosity induced by higher w/b values. Quantitative analysis reveals that the elastic modulus ratios (relative to the value at 60.0% leaching) at 10.0%, 30.0%, and 50.0% leaching degrees are 1.42, 1.25, and 1.08 for w/b = 0.4; 1.37, 1.22, and 1.07 for w/b = 0.5; and 1.47, 1.28, and 1.09 for w/b = 0.6. Notably, for D_c_ = 10.0%, 30.0%, and 50.0%, E at w/b = 0.40 exceeds that of w/b = 0.60 by 26.71%, 28.43%, and 30.28%, respectively, highlighting the pronounced impact of w/b on mechanical performance. Correspondingly, for a water–cement ratio of 0.4 and dissolution degrees of 0, 10%, 30%, 50%, and 60%, the porosity of concrete is 12.47%, 14.52%, 18.62%, 22.71%, and 24.76%, respectively; 16.63%, 18.34%, 21.77%, 25.21%, and 26.92% for w/b = 0.5; and 20.10%, 21.97%, 25.73%, 29.49%, and 31.37% w/b = 0.6. The mechanism of w/b on the elastic modulus of concrete can be explained as follows: The w/b governs porosity and hydration product density. Higher w/b values generate excess capillary pores from free water evaporation and less hydration products, reducing matrix compactness. In addition, these pores weaken aggregate–paste interfacial stiffness, diminishing load-transfer capacity and thus the elastic modulus of concrete. Conversely, lower w/b promotes dense hydration products (e.g., C-S-H gels), decreasing porosity and enhancing the modulus of concrete.

#### 4.2.2. The Influence of FA on E

**Figure 18:**
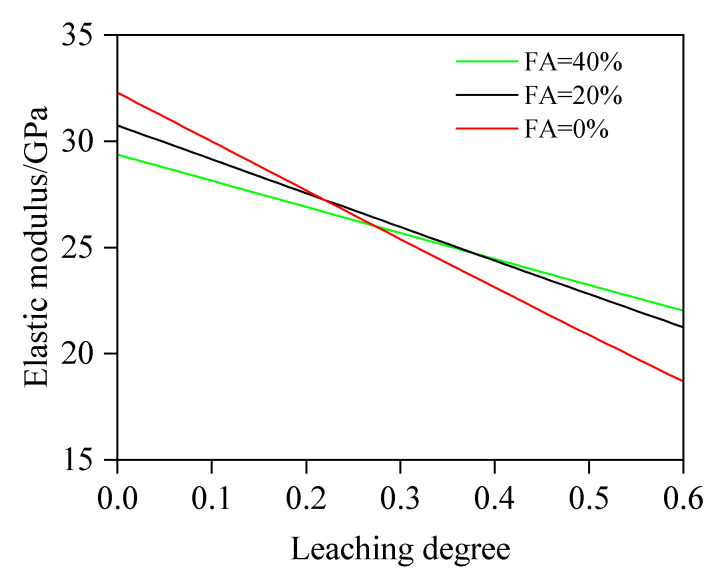
Effect of FA on E.

In this case, w/b is fixed at 0.5, and FA is 0, 20%, and 40%. The values of other variables are consistent with those in Section 4.2.1. Figure 18 shows the relationship between E and D_c_ at various FA values. The results show that fly ash concrete has a significantly slower reduction rate in the elastic modulus compared to plain concrete, which indicates that appropriate addition of fly ash will enhance the leaching resistance. With the elastic modulus at 60.0% leaching degree as the reference, the quantitative analysis yields a rate of 1.60, 1.35, and 1.11 times slower at 10.0%, 30.0%, and 50.0% leaching degrees, respectively, for FA = 0%; 1.37, 1.22, and 1.07 times slower, respectively, for FA = 20%; and 1.28, 1.16, and 1.05 times slower, respectively, for FA = 40%. Notably, for FA = 0%, 20%, and 40%, the decrease rates of elastic modulus are 22.7, 15.8, and 12.2 GPa.

#### 4.2.3. The Influence of *f_a_* on E

**Figure 19:**
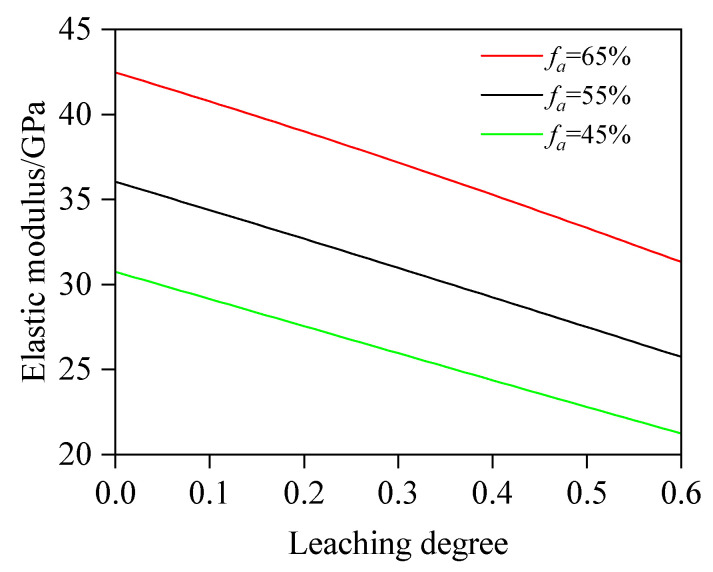
Effect of *f_a_* on E.

Regarding the influence of *f_a_* on E, w/b is fixed at 0.5, and *f_a_* is 45%, 55%, and 65%. The values of other variables are consistent with those in Section 4.2.1. Figure 19 shows the relationship between E and D_c_ at various *f_a_* values, where for a given degree of leaching, the elastic modulus increases with the rise in *f_a_*. Quantitative analysis shows the following: at concrete leaching degrees of 10.0%, 30.0%, and 50.0%, the elastic modulus of concrete with *f_a_* = 65% is 18.58%, 19.99%, and 21.17% higher, respectively, than that of concrete with *f_a_* = 55%; the elastic modulus of concrete with *f_a_* = 55% is 17.91%, 19.30%, and 20.59% higher, respectively, than that of concrete with *f_a_* = 45%; and the elastic modulus of concrete with *f_a_* = 65% is 39.82%, 43.15%, and 46.12% higher, respectively, than that of concrete with *f_a_* = 45%. From the simulation results, the variation regulation of the elastic modulus is sensitive to the volume fraction of the aggregate and exhibits a similar linear variation pattern.

#### 4.2.4. The Influence of ITZ Thickness on E

**Figure 20:**
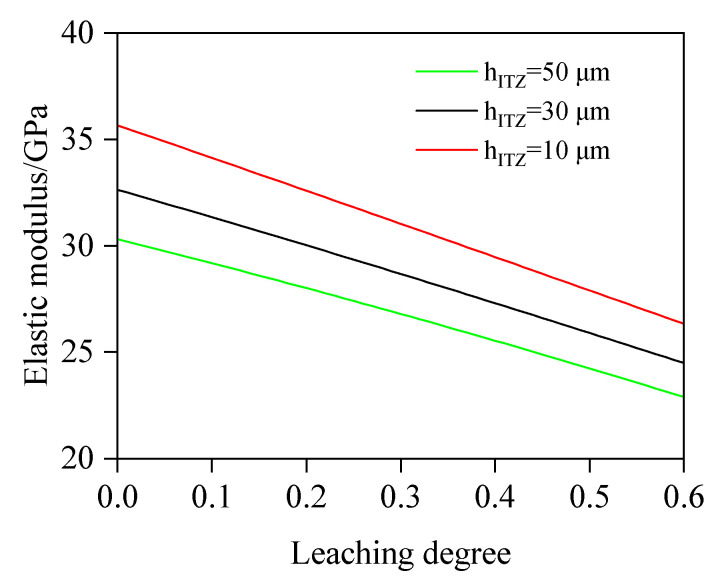
Effect of ITZ thickness on E.

In this case, w/b is fixed at 0.5, FA is 0%, and the ITZ thickness is 10, 30, and 50 μm. The values of other variables are consistent with those in Section 4.2.1. Figure 20 presents the relationship between E and D_c_ at different ITZ thicknesses. The results demonstrate that under identical leaching degrees, E decreases with an increasing ITZ thickness, primarily due to the higher porosity and lower elastic modulus in the ITZ. Quantitative analysis reveals that when h_ITZ_ decreases from 50 μm to 10 μm, E significantly increases by 16.96%, 15.80%, and 15.11% at 10.0%, 30.0%, and 50.0% leaching degrees, respectively. These findings highlight that reducing h_ITZ_ can effectively avoid the elastic modulus reduction induced by calcium leaching.

### 4.3. Suggestions for Practical Engineering

Based on the aforementioned analysis, four key strategies can be implemented in engineering practice to enhance concrete’s leaching resistance: optimizing the water-to-binder ratio, fly ash content, aggregate volume fraction, and ITZ compactness. First, a precisely controlled low water-to-binder ratio (recommended 0.35–0.45) significantly reduces capillary porosity by minimizing excess water, thereby densifying the microstructure and improving durability. Compared to the mix proportion with w/b = 0.5, when w/b is reduced to 0.3, the anti-leaching ability of fly ash concrete will increase by about 1.95 times. Second, increasing the volume fraction of well-graded aggregates (optimally 65–75%) while maintaining workability through a proper sand-to-aggregate ratio and particle size distribution enhances both the elastic modulus and leaching resistance. Compared to the mix proportion with f_a_ = 45%, when f_a_ is increased to 75%, the elastic modulus will increase by 58%. Third, incorporating 20–40% fly ash creates synergistic effects; their uniform dispersion during mixing fills micropores, refines the pore structure, and slows the degradation rate of C-S-H gels. Finally, ITZ optimization may be achieved by combining with 5–10% silica fumes to improve bonding, coupled with polycarboxylate-based superplasticizers to reduce water demand and ensure complete paste coating—collectively minimizing ITZ porosity and creating a more homogeneous composite matrix with superior leaching resistance.

## 5. Conclusions and Discussion

### 5.1. Conclusions

(1) This study proposes an elastic modulus prediction method for leached fly ash concrete considering the non-uniform model of the ITZ. By discretizing the ITZ into N equally thick spherical shell elements and combining chemical reaction equations to calculate porosity variations in these shells and the fly ash–cement bulk paste under different leaching degrees, the elastic modulus of leached cement paste and the N spherical shells is progressively computed based on the two-phase composite sphere model. Ultimately, the predictive model for the elastic modulus of leached fly ash concrete is established. The reliability of this model is validated through comprehensive comparisons with self-designed experimental data and third-party test results.
(2) Based on the developed computational model, quantitative analysis is conducted on the influence of key parameters (w/b, FA, *f_a_*, and h_ITZ_) on the elastic modulus. The results indicate the following: At leaching degrees of 10.0%, 30.0%, and 50.0%, specimens with w/b = 0.40 exhibit a 26.71%, 28.43%, and 30.28% higher elastic modulus, respectively, than those with w/b = 0.60, primarily due to a prominently elevated porosity induced by a higher w/b value. Quantitatively, at a leaching degree of 10.0%, the porosity of w/b = 0.60 is calculated as 21.97%, whereas that of w/b = 0.40 concrete is 14.52%. When leaching progresses to 30.0%, the porosities increase to 25.73% and 18.62%, respectively. At 50.0% leaching, porosity further climbs to 29.49% (w/b = 0.60) and 22.71% (w/b = 0.40). For FA = 0%, 20%, and 40%, the decrease rates of elastic modulus are 22.7, 15.8, and 12.2 GPa, respectively. The results show that fly ash concrete exhibits a significantly slower reduction rate in elastic modulus compared to plain concrete, which indicates that an appropriate addition of fly ash will enhance the leaching resistance. This is because pozzolanic reaction products exhibit superior resistance to calcium leaching compared to CH. The elastic modulus of concrete with *f_a_* = 65% is 39.82%, 43.15%, and 46.12% higher, respectively, than that of concrete with *f_a_* = 45%. From the simulation results, it can be found that the variation regulation of elastic modulus is sensitive to the volume fraction of the aggregate and exhibits a similar linear variation pattern. Regarding the impact of h_ITZ_, when h_ITZ_ decreases from 50 μm to 10 μm, E is significantly increased by 16.96%, 15.80%, and 15.11% at 10.0%, 30.0%, and 50.0% leaching degrees, respectively. These findings highlight that reducing h_ITZ_ can effectively avoid the elastic modulus reduction induced by calcium leaching.

### 5.2. Discussion About This Study and Future Work

The elastic modulus prediction model developed in this study is currently validated primarily for fly ash concrete systems. Its applicability remains constrained when multiple supplementary cementitious materials (e.g., slag) are incorporated concurrently. Moreover, the simulation methodology neglects the aggregate shape morphology. It should be noted that laboratory-accelerated leaching conditions diverge from actual field environments; indeed, natural leaching processes exhibit extreme slowness (typically several orders of magnitude slower than accelerated tests), modulated by temperature fluctuations and water chemistry. While the adopted accelerated leaching protocol enables the efficient simulation of degradation kinetics, it cannot fully replicate the complex natural exposure conditions or long-term degradation mechanisms. Consequently, model predictions require calibration against in situ monitoring data for engineering applications. Nevertheless, accelerated leaching research retains significant value due to the prohibitive time requirements for field validation. Finally, further research will focus on the limitations of predictive models, providing a scientific basis for structural maintenance and reinforcement.

## Figures and Tables

**Table 1:** Chemical constituents of cement and fly ash.

Material	CaO (%)	SiO_2_ (%)	Al_2_O_3_ (%)	Fe_2_O_3_ (%)	MgO (%)	SO_3_ (%)	Na_2_O (%)	K_2_O (%)	Loss of Ignition (%)
Cement	62.6	23.5	3.75	2.72	1.25	0.86	0.76	0.97	3.59
Fly ash	15.3	56.4	16.8	5.64	0.73	3.8	0.15	0.20	1.02

**Table 2:** Mixture proportions of concrete.

Group	FA	Fly Ash/(kg·m^−3^)	Cement/(kg·m^−3^)	Water/(kg·m^−3^)	Fine Aggregate/(kg·m^−3^)	Coarse Aggregate/(kg·m^−3^)	*f_a_*/%
A0	0%	0.00	660.56	336.4	654.23	548.41	45
A1	10%	66.17	595.54	330.86	654.23	548.41	45
A2	20%	130.22	520.88	325.55	654.23	548.41	45

**Table 3:** The depth of leaching, the degree of leaching, and elastic modulus of specimens with leaching time.

Group	Leaching Time/d	Leaching Depth/mm	Leaching Degree	Elastic Modulus/GPa
A0	3	2.82	0.110	30.71
	14	5.27	0.200	26.74
	28	7.48	0.277	26.09
	45	8.93	0.325	24.21
	90	12.25	0.430	22.07
A1	3	2.64	0.103	29.45
	14	4.57	0.174	28.80
	28	6.99	0.260	25.98
	45	8.50	0.311	24.33
	90	11.38	0.403	23.07
A2	3	2.33	0.091	29.09
	14	4.48	0.171	28.06
	28	6.56	0.245	26.44
	45	8.03	0.295	25.61
	90	10.81	0.386	24.86

**Table 4:** The values of variables.

w/b	FA	D_max_	D_min_	f_a_	h_ITZ_
0.4/0.5/0.6	20%	16.0	0.3	0.45	30 μm

## Data Availability

The original contributions presented in the study are included in the article, further inquiries can be directed to the corresponding authors.
